# The impact of subscribing to directors’ and officers’ liability insurance on corporate financialization: Evidence from China

**DOI:** 10.3389/fpsyg.2022.986135

**Published:** 2022-09-16

**Authors:** Cheng Peng, Wenting Fu, Xinyu Zhang, Hui Jiang

**Affiliations:** ^1^Center for International Business and Economy, School of International Finance and Trade, Sichuan International Studies University, Chongqing, China; ^2^School of Economics and Business Administration, Chongqing University, Chongqing, China

**Keywords:** directors’ and officers’ liability insurance, corporate financialization, risk-taking, financing constraints, audit quality, mediating mechanism

## Abstract

As an important corporate governance mechanism, directors’ and officers’ liability insurance is theoretically associated with corporate financialization because it directly affects incentive constraints and risk preference of enterprise managers. However, whether there is a causal relationship in fact has not been sufficiently empirically investigated. Using a sample of Chinese non-financial listed companies in Shanghai and Shenzhen A-shares from 2008 to 2020, this paper empirically analyzes how corporate subscription to directors’ and officers’ liability (D&O) insurance affects corporate financialization and examines the mediating role played by risk-taking, financing constraints, and audit quality. The study finds that corporate subscription to D&O insurance increases corporate financialization. In terms of the influential mechanism, subscription to D&O insurance promotes financialization by increasing risk-taking, alleviating financing constraints, and improving audit quality. In addition, the results in the heterogeneity analysis suggest that the promotion of financialization by subscribing to D&O insurance is more significant in state-owned enterprises, growth and decline stage enterprises, and non-dual-employment enterprises.

## Introduction

In recent years, the pressure of globalization, unfavorable competition among companies and the COVID-9 pandemic have led to a global economic downturn (Tijani et al., 2021). Along with the shrinking of market demand, overcapacity in the real economy has led to a significant decline in investment returns. However, profit margins in the financial and real estate sectors remain high, so a huge influx of capital into these industries with high yields has accelerated the expansion of China’s virtual economy. According to the statistical results of Wind Data, 767 listed companies in China’s A-shares purchased RMB 726.816 billion of wealth management products in 2016, while the number of listed companies purchasing wealth management products increased to 1,266 and the amount of wealth management products purchased exceeded to 1.28 trillion by 2021. However, Xu and Liu (2020) holds that physical capital is the main factor that affects the performance of a company. And faulty financial engineering, a focus on short-term profit, and a rush to consolidate are extremely dangerous from a financial viewpoint (Krulicky and Horak, 2021). With the increasing allocation of financial assets, the investment of real enterprises in the main business will be reduced accordingly, which will reduce the capital investment of the real economic sector, affect the fundamentals of China’s economic development, and cause people’s serious concern about “shifting from the real to the virtual” (Gu and Zhang, 2022; Liu et al., 2022; Zhang and Zhao, 2022). Therefore, what factors affect the financialization of enterprises and what are the internal causes of the allocation of financial capital have become the focus of discussion.

Corporate financialization has been a hot topic in academia in recent years, and many scholars have studied this issue from many different perspectives. From 2017 to the present, there are more than 200 articles indexed by SCI or SSCI in Web of Science alone in these years. The existing literature mainly studies the influencing factors of enterprise financialization from the following two perspectives. Firstly, macro factors, such as interest rate regulation (Yang et al., 2019), economic policy uncertainty (Peng et al., 2018), industrial policy (Guo et al., 2022), financial supervision (Ma and Yang, 2022), tax policy (Zhang et al., 2021), etc. Secondly, micro factors, such as corporate social responsibility (Liu et al., 2019), senior management background (Wang and Hu, 2022), information transparency (Liu et al., 2022), equity incentive (Li et al., 2021), etc. Although many scholars have studied the influencing factors of enterprise financialization from many aspects, only a small number of people have studied the behavior of enterprise financialization from the perspective of the behavior characteristics of enterprise executives. Actually, executives have the ability to make decisions that may affect the future of the enterprise (Valaskova et al., 2021a). As the decision-maker of enterprise asset allocation, senior executives have key control over the resources of the enterprise, which means that the personal behavior of senior executives will have a significant impact on the decision-making of enterprise financial asset allocation. Existing studies have found that the agency problem and overconfidence of managers will have a significant impact on the allocation of corporate financial assets (Zhang and Zhao, 2022). These studies confirm the dominant role of executive behavior characteristics in corporate financialization, and indirectly reveal the important impact of executive risk attitude. Consequently, it is very meaningful to explore the influence mechanism from the perspective of risk-taking of executives.

As a tool to hedge the risk of executive decision making, directors’ and officers’ liability insurance (D&O insurance) aims to provide protection against litigation risks arising from negligence and misconduct of directors and officers in the performance of their duties (Chen et al., 2016). U.S. listed companies have taken the lead in introducing D&O insurance to reduce the risks borne by directors and executives and to promote stable corporate development. Since China promulgated the “Guidelines For the Governance of Listed Companies” in 2002, which clearly stipulated the civil liability of senior executives of listed companies, Chinese listed companies introduced the D&O insurance for the first time. According to the purpose of D&O insurance, it aims to reduce the risk aversion tendency of senior executives to a certain extent by introducing a third-party institution to underwrite the decision-making mistakes of senior executives. To some extent, the allocation of financial assets in real enterprises is a high-risk behavior that deviates from the main business. Therefore, the subscription of directors’ liability insurance can have a significant impact on the financial asset allocation behavior of enterprises by changing the risk attitude of senior executives. Then, will the subscription of directors’ liability insurance significantly affect the financialization of enterprises? What’s the influence mechanism? So far, scholars have not paid enough attention to it.

This paper empirically analyzes the impact of subscribing to D&O insurance on firms’ financialization decisions by selecting a sample of Chinese A-share non-financial listed firms from 2008 to 2020, and examines the mediating roles played by risk-taking, financing constraints, and audit quality. In addition, this paper also analyzes the heterogeneous performance from the perspective of different property rights, different stage of life cycle and two jobs in one, respectively, in the further research. The possible contributions of this paper are: (1) Unlike previous studies about economic consequences of subscribing to D&O insurance, this paper examines its impact on corporate financialization, which enriches the understanding of the functions of D&O insurance and broadens the research on the influencing factors of enterprise financialization. (2) This paper examines the specific path of D&O insurance affecting the enterprise’s financialization, so as to put forward meaningful suggestions for the effective control of the enterprise’s financialization.

What’s more, this paper is structured as follows. The next section presents the theoretical analysis as well as the research hypotheses. Then the research design is described, including sample selection, data sources, variable measures, and model design, to empirically demonstrate the impact of subscribing to D&O insurance on firms’ financialization. In the final section, this paper discuss the empirical results and draw conclusions, and explore the potential implications of this study.

## Theory and research hypothesis

### D&O insurance and corporate financialization

D&O insurance, as a liability insurance policy, is purchased by a company or a joint venture and management for all directors, supervisors and officers to cover claims that may arise from decisions made in the course of their day-to-day duties. The existing literature shows that the main assumptions related to D & O insurance include incentive monitoring hypothesis and opportunism hypothesis. The incentive monitoring hypothesis suggests that the purchase of D&O insurance by directors and supervisors can reduce the risk they face in their work due to negligence or unintentional mistakes. Mitan et al. (2021) argue that managers must focus on the long-term implications for the future sustainability and profitability of their firms. Subscribing to D&O liability insurance not only reduces their attention to actively address the problems they face at work (Zhang and Xu, 2021), but also motivates them to operate boldly and innovate courageously, which can effectively contribute to the long-term sustainability of the firm. Studies have found that subscribing to D&O insurance helps to improve the business credit as well as credit rating of firms (Hu and Tan, 2018; Hu et al., 2019), and also significantly encourages the corporate to increase the level of risk-taking because to some extent the risks faced by the executives in the process of performing their duties would be transferred to the insurance institution (Wen, 2017). The opportunistic hypothesis, on the other hand, argues that D&O insurance isolates the insured’s personal wealth from the consequences of his or her actions and helps management hedge against risk. This tends to induce moral hazard and promote opportunistic behavior of management, which is equivalent to providing an umbrella for the executives. Subscribing to D&O insurance will transfer the risk of the management to the insurance company, thus reducing the self-interest cost of the management and further aggravating the agency conflict (Narjess et al., 2008). In other words, due to the protection of D&O insurance, the management might invest in some high-risk projects even if these projects would damage the interests of the enterprise (Feng et al., 2017).

In the environment of uncoordinated development of the industrial and financial industries, investing in financial assets can bring enterprises higher returns than their main business in the short term (Du et al., 2017), but it may cause harm to enterprises in the long term, which will make enterprises’ investment decisions deviate from the optimal behavior. This deviation from the optimization of investment decisions will bring the potential risk of being punished to the management. However, the purchase of D&O insurance will transfer the risk of management being punished to the insurance company, which may change the risk preference of enterprise management in financial investment. At first, because of the effect of “umbrella” of D&O insurance, executives may prefer to invest in much more financial assets in order to improve short-term performance of the firm (Du et al., 2017). Secondly, the risk underwriting effect of D&O insurance will reduce the management’s self-interest costs, which may induce their opportunistic behavior and promote short-term speculative behavior in investment decisions. Out of the pursuit of maximizing their own interests, the management may invest much more in financial assets with higher short-term yields (Gu and Zhang, 2022). Xu and Zeng (2011) found that enterprises usually have certain incentives for senior executives’ financial investment. According to the efficiency wage theory, the accounting profit of an enterprise will directly affect the income level of senior executives. Under the dual track operation mode of “entity + finance,” the income from financial assets is an important source of the accounting profit of an enterprise (Dai and Wang, 2021). Therefore, the higher the financial investment income, the higher the management’s salary will be. Consequently, due to the risk underwriting effect of D&O insurance, senior executives would have a stronger incentive to invest in financial assets.

Based on the above analysis, hypothesis H1 can be put forward: subscribing to D&O insurance will increase the corporate financialization.

### D&O insurance, risk-taking and corporate financialization

Business venture is considered to contribute to economic growth, innovation and technological progress (Duong et al., 2021), and 21st century jobs require leaders to be innovators and beneficiaries of risk, an attitude that a business should strive to change if it prefers a more stable, lower risk situation (Durana et al., 2020). Risk-taking can reflect a firm’s preferences when faced with risky decisions and the tendency to be willing to pay a price in order to obtain high profits (Yu et al., 2013). As the decision maker, the risk preference of executives has a critical impact on the risk-taking level of the company. Jensen and Meckling (1976) proposed the “management risk aversion” hypothesis, which states that the management is labor averse and risk averse. The risk averse executives are fearful of being sued in the process of performing their duties, which may lead to loss of growth opportunities. The introduction of D&O insurance reduces the litigation risk faced by executives on the one hand, and it can provide a protection for the management by covering the financial losses caused by the management’s decision failures. On the other hand, the underwriting effect of D&O insurance can also reduce the risk-averse tendency of managers and improve their risk-taking ability, as demonstrated by Hu and Hu (2017). While financial assets can yield high returns, they also result higher risks, including interest rate risk, legal risk, and market risk. Xing and Zhou (2020) found that subscribing to D&O insurance could enhance executives’ strategic aggressiveness. Therefore, D&O insurance would increase risk-taking preference of the firm, which enhances management’s willingness to accept risky projects, and thus promotes investment in financial assets and deepen financialization of firms.

Based on the above analysis, hypothesis H2 can be put forward: subscription of D&O insurance will facilitate corporate financialization by raising its level of risk-taking.

### D&O insurance, financing constraints and corporate financialization

External financing has a significant impact on the sustainability of a company. When faced with a high degree of financing constraints, it can easily lead to financial crises and hinder the investment of enterprises. According to financial theories, information asymmetry is an important influencing factor for enterprises to obtain the required financial support. While, D&O insurance can alleviate financing constraints from the following aspects, thus affecting enterprise investment and financialization. On the one hand, good corporate governance mechanisms help enterprises through a better access to financing and a lower cost of capital (Bulathsinhalage and Pathirawasam, 2017). Because the insurance claim probability of the D&O insurance is closely linked to the litigation risk faced by the insured company, when the internal governance of the enterprise is not very good, the insurance company will try to formulate stricter insurance terms to force the management to improve the level of corporate governance and the quality of financial reporting, thus reducing litigation risk (Lei et al., 2020). High level of corporate governance and a high-quality financial reporting will in turn help to assess the financial stability and solvency of a company (Valaskova et al., 2021c). On the other hand, in consideration of its own interests, the insurance company will investigate various basic information of the enterprise before signing the insurance agreement, and will continue to track the behavior of senior executives during the underwriting period. When the insured enterprise is facing litigation, it will investigate the case more carefully and comprehensively. Therefore, the insurance company will strengthen the external governance of the enterprise through the supervision before, during and after the event. The strengthening of external governance will send good information to the market, thus strengthening the confidence of the financial market in the enterprise, and finally weakening the financing constraints of the enterprise. In this scenario, the insured enterprise will be more likely to obtain low-cost funds from the market, thus providing more possibilities for enterprises to engage in financial asset investment (Peng et al., 2018). Therefore, it can be speculated that the subscription of D&O insurance by enterprises will ease the financing constraints, thus increasing the investment of enterprises in financial assets and deepening the financialization of enterprises.

Based on the above analysis, hypothesis H3 can be put forward: subscription of D&O insurance will facilitate corporate financialization by alleviating the financing constraints it faces.

### D&O insurance, audit quality and corporate financialization

High quality audit services represent a high degree of accuracy of statement information, credibility of financial information and consistency of audit conclusions with the real situation. In preparing financial statements, enterprises use various accounting methods, international or national accounting standards to manage the information on earnings obtained (Valaskova et al., 2021b). When enterprises choose high-quality audit services, corporate governance will be more standardized, information will be more reliable, and agency conflicts will be alleviated. When an enterprise chooses high-quality audit services, its governance will be more standardized, its information will be more reliable, its agency conflicts will be alleviated, and ultimately its financial risks and financing costs will be significantly reduced (Wang and Zhang, 2014; Zheng and Yan, 2016). The protective effect of directors’ liability insurance on the management will reduce the cost of aggressive actions taken by the management, thus increasing the company’s risky decisions and more opportunistic behavior. It has been shown that D&O insurance will lead to a reduction in the quality of financial information (Feng et al., 2017), and will increase the agency costs and litigation risks of insured enterprises (Yuan et al., 2018; Zeng and Li, 2018). Therefore, in order to protect their interests, shareholders have a strong incentive to promote the audit committee, which includes accounting professionals who do not have a direct relationship with the firm and have an obligation to safeguard the interests of the firm as a whole, to choose high-quality audit services (Hu et al., 2020). With the improvement of audit quality, the quality of accounting information provided by enterprises will be improved, so as to alleviate the information asymmetry between enterprises and fund providers, reduce the concerns of fund suppliers, and reduce their requirements for risk compensation, so that enterprises can obtain external funds at a lower cost, thus providing financial convenience for enterprise financial asset investment. On the other hand, the improvement of audit quality can alleviate the information asymmetry between shareholders and managers, strengthen the internal governance mechanism of the enterprise, and the financial investment plan proposed by the management is easier to reach a consensus on decision-making, so as to facilitate the decision-making of financial asset investment and promote the financialization of the enterprise.

Based on the above analysis, hypothesis H4 can be put forward: subscription of D&O insurance will facilitate corporate financialization of by improving audit quality.

## Research methods

### Data and sample

The samples in this paper are non-financial listed companies in China A-shares from 2008 to 2020, and are selected according to the following rules: (1) exclude the samples with incomplete key data; (2) exclude the samples of ST and *ST listed companies, and finally obtain 28,741 valid observation samples. Regarding the source of samples, the sample of D&O insurance used in this paper is from the Chinese CNDRS database, and the data related to other variables such as corporate financialization and risk-taking are from the Chinese CSMAR and WIND databases.

### Definition of variables

#### Dependent variables

Corporate Financialization (*Fin*): referring to the method of Wan et al. (2011), the degree of corporate financialization is measured using the proportion of financial assets held by enterprises to total assets. Financial assets held by enterprises include: derivative financial assets, net available-for-sale financial assets, net investment properties, net trading financial assets, net loans and advances granted, and net hold investment due.

#### Independent variables

D&O Insurance (*Doi*): generally speaking, the measurement of directors’ executive liability insurance can usually be divided into the following three types: (1) recognition criteria of the two-class method: dummy variables of 0 and 1 are used to measure based on whether or not directors’ executive liability insurance is purchased; (2) recognition criteria of absolute amount: specific values of premiums or coverage are used as the measurement criteria; (3) recognition criteria of relative amount: the ratio of the policyholder’s book to all executives of D&O insurance is used as the criterion. However, since it is not mandatory in China for listed companies to disclose the information of D&O insurance subscription, this paper adopts the method of 0 and 1 dummy variables commonly used by domestic and foreign scholars to measure.

#### Mediating variables

Risk-taking (*Risk*): this paper draws on Yu et al. (2013) to choose the volatility of corporate earnings to measure the level of risk-taking. *ROA_i_* is the ratio of earnings before taxes, interest, depreciation and amortization (*EBITDA*) to the total assets of the company in year *i*. The volatility is calculated by first adjusting the *ROA* for each year using the industry average, and then calculating the standard deviation of the industry-adjusted *ROA* for each observed period of time, using the following formula.


$$\mathrm{Risk}T_{i}=\sqrt{\frac{1}{N-1}\overset{N}{\underset{n=1}{\sum }}{\left(ADJ\_ROA_{\mathrm{in}}-\frac{1}{N}\overset{N}{\underset{n=1}{\sum }}ADJ\_ROA_{\mathrm{in}}\right)}^{2}}|N=3\text{,}$$

$$ADJ\_ROA_{\mathrm{in}}=\frac{\mathrm{EBITD}A_{\mathrm{in}}}{\mathrm{ASSET}S_{\mathrm{in}}}-\frac{1}{X_{n}}\overset{N}{\underset{k=1}{\sum }}\frac{\mathrm{EBITD}A_{kn}}{\mathrm{ASSET}S_{kn}}$$

Financing constraints (*FC*): Chinese existing literature on the measurement of financing constraints mainly uses the *SA* index, *KZ* index, *WW* index, and *FC* index. Drawing on the approach of Gu et al. (2020), this paper choose to construct an *FC* index to measure the financing constraint, which is calculated as follows. First, the three variables of firm size, cash dividend payout ratio, and age are standardized by year, and the mean value of the treated variables is taken to determine the financing dummy variable *QUFC.* Firms with mean values above the one-third quantile are defined as the light financing constraint group *QUFC* is assigned to 0, and firms with mean values below the one-third quantile are defined as the heavy financing constraint group *QUFC* is assigned to 1. The Logit model is then used to simulate the probability of occurrence of the firm’s annual financing constraint, which is defined as the financing constraint index *FC* (taking values between 0 and 1), with larger *FC* values representing the higher degree of financing constraint faced by the firm, and the model used is shown below:

$P\left(\mathrm{QUFC}=1|Z_{i,t}\right)=e^{Z_{i,t}}/(1+e^{Z_{i,t}}$ )


$$\begin{array}{c} Z_{i,t}=\theta_{0}+\theta_{1}size_{i,t}+\theta_{2}lev_{i,t}+\theta_{3}{\left(\mathrm{CASHDIV}/ta\right)}_{i,t}\\ \;+\theta_{4}MB_{i,t}+\theta_{5}{\left(NWC/ta\right)}_{i,t}+\theta_{6}{\left(\mathrm{EBIT}/ta\right)}_{i,t} \end{array}$$

where *CASHDIV* represents cash incentives declared for the year, *ta* represents total assets, *NWC* represents net working capital, and *EBIT* represents earnings before interest and taxes.

Audit quality (*Infee*): most scholars believe that the quality of audit services is positively related to audit fees, and that the level of audit fees reflects, to a certain extent, the goodness of the firm, the effort expended by the accountant and the quality of services. Therefore, this paper draws on the study by Ye et al. (2020) to use the amount of audit fees to measure audit quality while taking the natural logarithm of it.

#### Control variables

Referring to the related literature (Gao et al., 2021; Tang et al., 2021), this paper incorporates other relevant variables that may affect the role of director’s officer liability insurance on corporate financialization into the model of this paper. These variables include: *top1、bm、mfee、lev、growth、roa、ato、balance* and *indep.* Based on the order presented in the regression results below, the definition of each control variable and the predicted impact on the model are as follows.

Top shareholder ownership ratio (*top1*): number of shares held by the first largest shareholder/total number of shares. Generally speaking, the higher the shareholding of the first largest shareholder, the greater the incentive the majority shareholder may have to work hard to operate the business to enhance the core competencies of the company, which in turn will enhance the future performance of the company. As a result, companies will invest less in financial arbitrage projects and more in long-term operational investments such as physical assets and R&D and innovation projects, which in turn will increase the long-term value of the company. This predicts that the regression coefficient of the control variable *top1* should be negative.

Book-to-market ratio (*bm*): book value/total market value. A high book-to-market ratio indicates that the listed company has a high investment value in the stock market, and this type of company tends to receive more attention from the outside world in the stock market, which in turn exerts a stronger external monitoring pressure on the company. This pressure to some extent inhibits management’s over-investment in financial assets for self-interest motives. Therefore, it can be predicted that the regression coefficient of the control variable *bm* should be negative.

Management expense ratio (*mfee*): management expenses divided by operating income. A higher management expense ratio represents a less efficient management of the firm, which may exacerbate management’s short-sighted behavior as well as inefficient investments that tend to hold more financial assets to obtain high returns and underestimate their potential risks. Therefore, the regression coefficient of the control variable *mfee* should be positive.

Gearing ratio (*lev*): the ratio of total assets to total liabilities. A higher gearing represents a relatively higher financial risk for the firm, which not only reduces the availability of bank credit, but also affects the availability of corporate finance. This will make enterprises’ investment more cautious and further inhibit their investment in high-risk financial products. Therefore, the coefficient of variable *lev* should be negative.

Corporate growth (*growth*): business revenue growth rate. The stronger the growth of an enterprise means the stronger the sustainability of the enterprise in the future, and the management of this type of enterprises is less short-sighted and will pay more attention to long-term investment, thus playing a certain inhibiting effect on the financialization of enterprises. Thus, the regression coefficient of the variable *growth* is predicted to be negative.

Total asset margin (*roa*): net income/average total assets. A higher utilization rate of total assets means a more profitable enterprise, a more efficient utilization of assets and a higher level of management. On one hand, high profit means the enterprises can mobilize much more free cash flow to invest whatever they want to invest. On the other hand, the higher this indicator is, the easier it is for the enterprises to obtain bank loans. Consequently, enterprises have a strong incentive to invest these cheap funds and then obtain high returns. Therefore, it can be inferred that the regression coefficient of the variable *roa* should be positive.

Total asset turnover ratio (*ato*): ratio of operating income to total assets. A higher asset turnover ratio reflects a company’s ability to operate its assets, which means that the company has a strong sales capacity and a more considerable income from its main business. To a certain extent, this will make enterprises invest more in their main business and less in financial products. Therefore, the coefficient of the variable *ato* should be negative.

Equity balance (*balance*): the sum of the shareholdings of the second to fifth largest shareholders divided by the shareholding of the first largest shareholder. Equity balances means that control power is shared among several major shareholders, and it is impossible for any one major shareholder to control the decisions of the listed company alone. The higher the degree of equity balances, the more effective it will be in inhibiting the infringement of the interests of listed companies by major shareholders, which in turn will promote the enhancement of corporate value, increase corporate investment in long-term business projects such as physical assets and innovative projects, and enhance the sustainable business ability of enterprises. This predicts that the coefficient of the variable *balance* should be negative.

Independent director ratio (*indep*): independent directors divided by the number of directors. Independent directors usually put forward reasonable suggestions on the decision-making of the enterprise according to their professional expertise, so as to reduce the inefficient investment of the enterprise. In order to avoid joint and several liabilities caused by the company’s decision-making mistakes, the independent directors would try their best to provide prudent and scientific suggestions. Therefore, independent directors will reduce the possibility of financial investment. From this, it is predicted that the coefficient of the variable *indep* should be negative.

This paper also controls for *industry* and *year* variables. See Table 1 for the definition of variables and measurement indexes used in this paper.

**Table 1:** Definition of variables and measurement indexes.

Variable types	Variable	Measurement
Dependent variable	Corporate Financialization (*Fin*)	By using the proportion of financial assets held by enterprises to total assets.
Independent variable	D&O insurance(*Doi*)	If the current listed company purchases directors’ liability insurance takes the value of 1, otherwise it is 0.
Mediating variables	Risk-taking(*Risk*)	Volatility of company earnings.
	Financing constraints (*FC*)	Construction of FC index.
	Audit quality (*Infee*)	Natural logarithm of audit fees within the firm.
Control variables	Top shareholder ownership ratio (*top1*)	Number of shares held by the first largest shareholder/total number of shares.
	Book-to-market ratio (*bm*)	Book value/total market value.
	Management expense ratio (*mfee*)	Management expenses divided by operating income.
	Gearing ratio (*lev*)	The ratio of total assets to total liabilities.
	Corporate growth (*growth*)	Business revenue growth rate.
	Total asset margin (*roa*)	Net income/average total assets.
	Total asset turnover ratio (*ato*)	Ratio of operating income to total assets.
	Equity balance (*balance*)	The sum of the shareholdings of the second to fifth largest shareholders divided by the shareholding of the first largest shareholder.
	Independent director ratio (*indep*)	Independent directors divided by the number of directors.
	*Industry*	Control
	*Year*	Control

### Model construction

This paper construct model (1) to test the aggregate effect of D&O insurance on the impact of corporate financialization:


(1) $$Fin_{\mathrm{it}}=\alpha_{0}+\alpha_{1}\mathrm{DO}I_{\mathrm{it}}+\alpha_{2}\sum \mathrm{Control}s_{\mathrm{it}}+\delta_{i}+\delta_{t}+\varepsilon_{\mathrm{it}}$$

where *Fin* represents the degree of financialization of the firm, *DOI* represents whether the firm subscribes to D&O insurance, *Controls* represents the control variables, and represent year and industry fixed effects and represents the residual term. If is greater than 0, it means that purchasing director liability insurance will increase the financialization of the firm, and if it is less than 0, it means that purchasing director liability insurance will inhibit the financialization of the firm. At the same time, to investigate the mediating role of risk-taking, financing constraints and audit quality, models (2, 3) are constructed to test them:


(2) $$\mathrm{Channe}l_{\mathrm{it}}=\beta_{0}+\beta_{1}\mathrm{DO}I_{\mathrm{it}}+\beta_{2}\sum \mathrm{Control}s_{\mathrm{it}}+\delta_{i}+\delta_{t}+\varepsilon_{\mathrm{it}}$$

(3) $$\begin{array}{c} Fin_{\mathrm{it}}=\gamma_{0}+\gamma_{1}\mathrm{DO}I_{\mathrm{it}}+\gamma_{2}\mathrm{Channe}l_{i,t}+\gamma_{3}\sum \mathrm{Control}s_{\mathrm{it}}\\ \;+\delta_{i}+\delta_{t}+\varepsilon_{\mathrm{it}} \end{array}$$

where *Channel* is the general name of the selected mediating variables, and model (2) is a test of the effect of D&O insurance on the mediating variables. Model (3) is a test of the aggregate effect between the D&O insurance, intermediary variables, and the financialization of the firm. In this paper, a three-step approach is used to test the mediation mechanism, and the main steps are as follows.

In the first step, model (1) is first tested and, if $\alpha_{1}$ is significant, the next step is tested.

In the second step, the direct and indirect effects of the mediation model are tested by regressing model (2) and model (3) separately, and if both are significant, it means that the indirect effect exists and proceeds to the next test. If at least one coefficient is insignificant, the confidence interval is tested using Bootstrap, and if significant, the next step is performed, and if insignificant, the mediating effect does not exist.

In the third step, it is judged whether $\gamma_{1}$ is significant, and if so, the direction of the sign of $\gamma_{1}$ and $\beta_{1}\gamma_{2}$ should be judged. The same sign indicates that there is a partial mediation effect, while the opposite sign indicates that there is a masking effect.

## Empirical results

### Descriptive statistics

Table 2 reports the results of descriptive statistics for the main variables. The sample mean for D&O insurance (*DOI*) is 0.0688, indicating that 6.9% of the observed sample of listed companies subscribe to D&O insurance, the percentage that remains largely consistent with the findings of Gao et al. (2021). Through further annual analysis of this indicator, it is found that, by the end of 2019, nearly 400 enterprises have subscribed for D&O insurance, and in 2020, there were 170 new companies insured by D&O insurance, the increase is nearly half of the stock. However, no more than 100 listed companies have subscribed to D&O insurance before 2012, which indicates that the rate of D&O insurance coverage for listed companies in China is rising rapidly in these years.

**Table 2:** Descriptive statistics.

Variables	Number	Mean	Standard deviation	Min	Max
Fin	28,231	0.0369	0.0714	0	0.405
DOI	28,231	0.0691	0.254	0	1
top1	28,231	0.349	0.149	0.0865	0.748
bm	28,231	1.062	1.120	0.101	6.706
mfee	28,231	0.0878	0.0672	0.00910	0.402
lev	28,231	0.435	0.204	0.0558	0.884
growth	28,231	0.396	1.075	−0.650	7.779
roa	28,231	0.0373	0.0593	−0.243	0.193
ato	28,231	0.628	0.430	0.0778	2.527
balance	28,231	0.700	0.602	0.0250	2.770
indep	28,231	37.39	5.350	30.77	57.14

### Correlation analysis

In this paper, a preliminary correlation analysis of the relevant variables designed by the main effect model (1) was conducted using Stata16, and the results of the analysis are shown in Table 3. According to the correlation analysis results, it can be tentatively concluded that the subscription of corporate D&O insurance will increase the degree of corporate financialization, which verifies the reasonableness and feasibility of hypothesis H1 in this paper to a certain extent, but further regression test analysis is needed.

**Table 3:** The results of correlation analysis.

Variables											
*Fin*	1.000										
*DOI*	0.013^**^	1.000									
*top1*	−0.019^***^	0.024^***^	1.000								
*bm*	−0.029^***^	0.176^***^	0.108^***^	1.000							
*mfee*	0.066^***^	−0.069^***^	−0.165^***^	−0.291^***^	1.000						
*lev*	−0.090^***^	0.110^***^	0.067^***^	0.569^***^	−0.294^***^	1.000					
*growth*	0.024^***^	−0.005	0.018^***^	0.064^***^	0.058^***^	0.095^***^	1.000				
*roa*	0.022^***^	−0.030^***^	0.128^***^	−0.219^***^	−0.154^***^	−0.352^***^	−0.011^*^	1.000			
*ato*	−0.097^***^	0.019^***^	0.083^***^	−0.010^*^	−0.417^***^	0.141^***^	−0.160^***^	0.115^***^	1.000		
*balance*	−0.017^***^	0.012^*^	−0.683^***^	−0.093^***^	0.105^***^	−0.136^***^	−0.015^**^	−0.003	−0.078^***^	1.000	
*indep*	0.041^***^	0.018^***^	0.040^***^	0.013^*^	0.044^***^	−0.017^***^	0.019^***^	−0.018^***^	−0.041^***^	−0.019^***^	1.000

* *p* < 0.1;

** *p* < 0.05;

*** *p* < 0.01.

### Regression result analysis

This paper uses OLS method to estimate the effect of D&O insurance subscription on corporate financialization and controls for industry effects as well as year effects in the model, and the regression results are shown in Table 4.

**Table 4:** The results of regression analysis.

	(1)	(2)	(3)	(4)	(5)	(6)	(7)
Variables	*Fin*	*Risk*	*Fin*	*FC*	*Fin*	*Infee*	*Fin*
*DOI*	0.005^***^	0.002^*^	0.005^***^	−0.078^***^	0.003^*^	0.479^***^	0.005^***^
	(3.421)	(1.770)	(3.399)	(−16.632)	(1.679)	(9.34)	(3.228)
*Risk*			0.017^**^				
			(2.067)				
*FC*					−0.036^***^		
					(−17.691)		
*Infee*							0.001^***^
							(3.384)
*top1*	−0.022^***^	−0.008^***^	−0.021^***^	−0.169^***^	−0.028^***^	0.497^***^	−0.022^***^
	(−5.680)	(−2.743)	(−5.645)	(−15.275)	(−7.290)	(4.086)	(−5.761)
*bm*	−0.002^***^	−0.004^***^	−0.002^***^	−0.064^***^	−0.004^***^	0.224^***^	−0.002^***^
	(−3.396)	(−11.269)	(−3.250)	(−46.050)	(−7.973)	(14.596)	(−3.677)
*mfee*	0.023^***^	0.049^***^	0.022^***^	0.214^***^	0.030^***^	0.763^***^	0.022^***^
	(2.973)	(8.911)	(2.859)	(9.704)	(4.005)	(3.147)	(2.910)
*lev*	−0.043^***^	0.000	−0.043^***^	−0.694^***^	−0.068^***^	0.819^***^	−0.043^***^
	(−15.700)	(0.104)	(−15.702)	(−87.549)	(−22.178)	(9.402)	(−15.868)
*growth*	−0.003^***^	−0.001^***^	−0.003^***^	0.012^***^	−0.003^***^	−0.059^***^	−0.003^***^
	(−8.006)	(−2.896)	(−7.970)	(10.302)	(−6.952)	(−4.651)	(−7.911)
*roa*	0.020^***^	−0.215^***^	0.023^***^	−0.546^***^	0.000	1.419^***^	0.019^**^
	(2.567)	(−38.733)	(2.966)	(−24.474)	(0.002)	(5.787)	(2.449)
*ato*	−0.014^***^	0.005^***^	−0.015^***^	0.024^***^	−0.014^***^	0.244^***^	−0.015^***^
	(−12.423)	(5.474)	(−12.485)	(6.944)	(−11.750)	(6.536)	(−12.548)
*balance*	−0.010^***^	0.002^***^	−0.010^***^	−0.014^***^	−0.010^***^	0.036	−0.010^***^
	(−10.320)	(3.492)	(−10.361)	(−5.064)	(−10.905)	(1.212)	(−10.346)
*indep*	0.000^***^	−0.000	0.000^***^	0.000^*^	0.000^***^	−0.001	0.000^***^
	(3.913)	(−0.043)	(3.913)	(1.769)	(4.120)	(−0.502)	(3.923)
*Cons*	0.051^***^	0.075^***^	0.050^***^	0.958^***^	0.085^***^	10.731^***^	0.044^***^
	(9.558)	(19.520)	(9.256)	(61.697)	(15.123)	(62.865)	(7.766)
							
*N*	28,231	28,231	28,231	28,231	28,231	28,231	28,231
*R^2^*	0.139	0.216	0.139	0.509	0.148	0.166	0.139
*F*	108.129	184.928	105.726	694.539	114.062	133.704	105.920
*Industry*	control	control	control	control	control	control	control
*Year*	control	control	control	control	control	control	control

*t* statistics in parentheses.

* *p* < 0.1;

** *p* < 0.05;

*** *p* < 0.01.

Column (1) presents the main effect analysis of subscription to D&O insurance on the degree of financialization of the firm. The regression results show that the coefficient of DOI is 0.005 and significant at the 1% level, indicating that subscription to D&O insurance deepens the financialization of the firm, taking into account the time effect and industry characteristics. In addition, except for *indep*, the direction of other control variable coefficients is consistent with expectations. The negative sign of *indep* shows that, on the one hand, the supervisory function of independent directors in China is not fully played. On the other hand, it manifests that because of protection of D&O insurance, the independent directors are largely exempted from the joint and several liabilities arising from decision-making mistakes, which makes them not only not hinder the company from making short-term financial project investment, but also make use of their own unique professional ability or resources to provide advice for the enterprise to make financial project investment, so as to win higher returns for the enterprise. Therefore, independent directors will positively promote financial investment of enterprises.

Columns (2, 3) are regression results of the mediating mechanism of risk-taking. The regression results in column (2) show that the coefficient of DOI is 0.002 and significantly positive at the 10% level, which indicates that firms’ subscription to D&O insurance increases their risk-taking preference, in line with the previous hypothesis. The coefficient of DOI in column (3) is 0.005 (significant at the 1% level) and the coefficient of Risk is 0.017 (significant at the 5% level), so the indirect effect exists according to the analysis of the mediation test process. Because signs of $\gamma_{1}$ and $\beta_{1}\gamma_{2}$ are in the same direction, the results verify the existence of a partial mediating effect, suggesting that subscribing to D&O insurance deepens corporate financialization by increasing the level of risk-taking, consistent with the previous hypothesis H2.

Columns (4, 5) are tests for the mediating mechanism of financing constraints. In column (4), the regression coefficient of DOI is-0.078 (significant at the 1% level), indicating that firms’ subscription to D&O insurance alleviates their financing constraints, which is consistent with the prediction of this paper. The coefficients of DOI and FC in column (5) are 0.003 and-0.036, which are significant at the 10 and 1% levels, respectively, and by judging the direction of the sign of $\gamma_{1}$ and $\beta_{1}\gamma_{2}$ , the financing constraint also plays a partial mediating effect, indicating that subscription to D&O insurance will promote the degree of financialization of firms by alleviating the financing constraint they face, proving hypothesis H3.

Columns (6, 7) are tests of audit quality as a mediating mechanism. The regression results in column (6) show that the coefficient of DOI is significantly positive at the 1% level, indicating that subscribing to D&O insurance enhances audit quality of firms. The coefficients of DOI and Infee in column (7) are 0.005 and 0.001, respectively, and both are significant at the 1% level, which proves the existence of the mediating effect. And from the coefficients of these three, it can be judged that audit quality plays a partial mediating effect, which verifies the hypothesis H4 of this paper: subscribing to D&O insurance will promote the financialization of firms by improving audit quality.

### Robustness tests

#### Propensity score matching

In this paper, the propensity score matching (PSM) method was used to control the endogeneity problem. In this paper, based on the event of whether a firm purchases D&O insurance or not, firms in the sample that subscribe to D&O insurance are used as the treatment group and firms that do not purchase D&O insurance are used as the control group, while the control variables involved in the above are used as matching variables. The basic logic of PSM is to calculate comprehensive scores for multiple factors affecting individual characteristics, and use this as a criterion to match samples with similar scores in the treatment and control groups to achieve a random grouping. In this paper, 1:1 nearest neighbor matching is chosen to process the sample of this paper. First, a logit model is applied to score the propensity of whether the listed companies in the sample subscribe to D&O insurance, i.e., companies that subscribe to D&O insurance are matched with companies that do not subscribe to D&O insurance, and then the matched sample is regressed.

The reliability of PSM depends on whether there is a significant difference between the observed variables of the treatment and control samples after matching, and if the difference is significant, it indicates that the observed variables or the matching method is inappropriate. Therefore, it is necessary to perform a balance test on the matched feature vectors to observe whether they satisfy the balance hypothesis, and the results of the balance test are shown in Table 5. the results in the table show that the standard deviations of most of the variables in the treatment and control groups are significantly reduced after matching, and the absolute values of the standard deviations after matching are less than 10%, and there is no significant difference between the feature variables in the treatment and control groups after t-test, so can indicate that the results after PSM matching are reliable. The regression of the PSM-treated samples (the regression results are shown in Table 6) shows that the regression coefficient of DOI is significantly positive, indicating that the promotion effect of subscribing to D&O insurance on corporate financialization is robust, and this result supports the hypothesis of this paper.

**Table 5:** Matching variable balance test results.

Variables	Matched or not	Mean		Standard deviation (%)	Reduction of standard deviation (%)	*T*-test	
		Treated	Control			*T*-Value	*p*-Value
*top1*	Unmatched	0.36243	0.34831	9.4	85.5	4.03	0.000
	Matched	0.36243	0.36447	−1.4		−0.41	0.684
*bm*	Unmatched	1,7,866	1.0079	55.9	94.7	30.08	0.000
	Matched	1.7866	1.7457	2.9		0.76	0.448
*mfee*	Unmatched	0.7086	0.08908	−28.7	99.8	−11.58	0.000
	Matched	0.7086	0.07091	−0.1		−0.02	0.982
*lev*	Unmatched	0.51791	0.42934	44.2	92.1	18.60	0.000
	Matched	0.51791	0.52495	−3.5		−1.07	0.284
*growth*	Unmatched	0.37422	0.39737	−2.2.	−3.0	−0.92	0.359
	Matched	0.37422	0.35037	2.2		0.74	0.460
*roa*	Unmatched	0.03081	0.03781	−12.0	68.1	−5.03	0.000
	Matched	0.03081	0.02858	3.8		1.23	0.218
*ato*	Unmatched	0.6588	0.62592	7.0	49.5	3.26	0.001
	Matched	0.6588	0.67542	−3.5		−1.04	0.299
*balance*	Unmatched	0.72697	0.69749	5.0	86.6	2.09	0.037
	Matched	0.72697	0.73093	−0.7		−0.20	0.842
*indep*	Unmatched	37.738	37.361	7.0	75.5	3.01	0.003
	Matched	37.738	37.831	−1.7		−0.51	0.610

**Table 6:** Regression results of the sample after PSM treatment.

	After PSM treatment:*Fin*
*DOI*	0.006^***^
	(2.76)
Cons	0.040^***^
	(2.76)
Control variables	control
Year	control
Industry	Control
*N*	3,900
*R^2^*	0.1747
*F*	19.91

*p < 0.1;

**p < 0.05;

***p < 0.01.

#### Heckman two-stage regression

To avoid possible sample selection bias, this paper uses the Heckman two-stage method to test the baseline regression results. This paper uses the mean value of other companies in the same industry that have subscribed to D&O insurance (MDOI) as an exogenous instrumental variable because the more companies in the same industry purchase D&O insurance the more likely it is that the company will be stimulated to subscribe to D&O insurance, while the purchase of D&O insurance by other companies in the same industry does not have a direct impact on the company and meets the relevant requirements for selecting instrumental variables. In the first stage of Heckman’s regression, this paper sets DOI as the explanatory variable, adds MDOI to the regression using the Probit model, and obtains the inverse Mills ratio (IMR) using the regression results of the first stage. In the second stage, the inverse Mills ratio (IMR) was added into the Heckman second stage model for fitting, and the results of the two-stage regression are shown in Table 7. The regression result show that the coefficient of DOI is still significantly positive at the 1% level, and in order to avoid the problem of multicollinearity caused by the inclusion of IMR, VIF test was conducted in this paper after the second-stage regression, and the results show that the VIFs of all variables do not exceed 10, so the conclusions of this paper can be proved to be robust.

**Table 7:** Results of the Heckman two-stage method.

	First-stage		Second-stage
	*DOI*		*Fin*
*MDOI*	−8.424^***^	DOI	0.005^***^
	(−11.90)		(3.202)
		IMR	−0.008^***^
			(−2.759)
*top1*	0.304^***^	top1	−0.024^***^
	(2.65)		(−6.136)
*bm*	0.181^***^	bm	−0.003^***^
	(15.38)		(−4.394)
*mfee*	−0.078	mfee	0.026^***^
	(−0.31)		(3.361)
*lev*	0.487^***^	lev	−0.046^***^
	(5.75)		(−14.991)
*growth*	−0.023^*^	growth	−0.003^***^
	(−1.87)		(−7.496)
*roa*	0.089	roa	0.019^**^
	(0.38)		(2.498)
*ato*	0.145^***^	ato	−0.015^***^
	(4.39)		(−12.594)
*balance*	0.152^***^	balance	−0.011^***^
	(5.45)		(−10.542)
*indep*	0.006^***^	indep	0.000^***^
	(2.66)		(3.509)
*_cons*	−2.453^***^	_cons	0.075^***^
	(−14.02)		(7.045)
Year	control	Year	control
Industry	control	Industry	control
*N*	28,101	*N*	28,101
*R* ^2^	0.0950	*R* ^2^	0.139

** *p* < 0.1;

** *p* < 0.05;

*** *p* < 0.01.

#### Placebo test

This paper draws on Chen et al. (2021) to use placebo tests to exclude omitted variables from confounding the paper’s findings. First, whether or not to subscribe to D&O insurance is randomly swapped among listed companies and regressed using the re-matched data. The results are shown in Table 8 Panel A. If the contribution of subscription to D&O insurance to corporate financialization is due to omitted variables, then the coefficient of DOI will remain significantly positive after re-matching. However, the negative randomly transformed DOI coefficient in Panel A in the table contradicts the findings of this paper, suggesting that the promotion effect of subscription to directors’ insurance on corporate financialization is not due to omitted variables.

**Table 8:** Robustness test results.

	Panel A	Panel B	Panel C
*DOI*	−0.003^*^	0.003^***^	0.043^***^
	(−1.83)	(2.66)	(6.94)
Cons	0.050^***^	0.035^***^	0.245^***^
	(9.35)	(8.61)	(6.89)
Control variables	control	control	control
Year	control	control	control
Industry	control	control	control
*N*	28,231	28,231	24,264
*R^2^*	0.1381	0.0832	0.1264
*F*	107.54	60.89	94.70

*p < 0.1;

**p < 0.05;

***p < 0.01.

**Figure 1:**
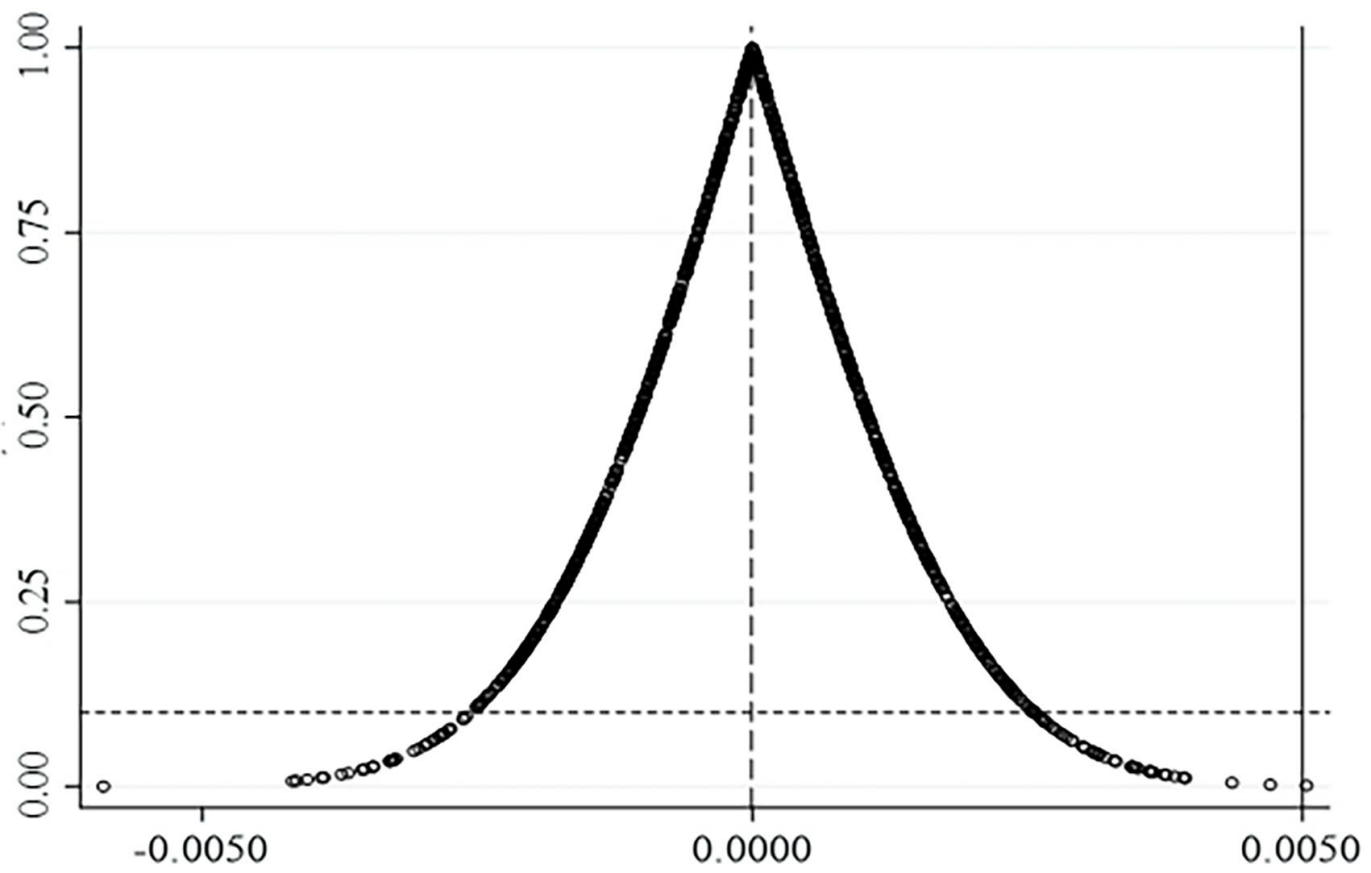
The result of placebo test. The horizontal axis represents the coefficient and the vertical axis represents the value of *p*.

To further enhance the robustness of the placebo results, the above steps were regressed 1,000 times repeatedly in this paper and the regression coefficients and *p*-values after statistics were plotted in Figure 1. As can be seen from the scatter plot in Figure 1, the number of regressions satisfying a positive coefficient and a value of p less than 0.1 in these 1,000 regressions is very small, indicating that the virtual treatment effect constructed in this paper does not exist proving the robustness of the conclusions of this paper.

#### Alternative explained variable

Considering that investment property is weaker than other financial assets in terms of liquidity, and the government’s policy orientation has enhanced the entity attribute of real estate, this paper refers to the practice of Liu and Xie (2021) to delete investment property from the sub-indicators of Fin in the previous paper, and use the remaining five sub-indicators of financial assets as a measure of corporate financialization (Fin2) for regression test, and the results are shown in Table 8 Panel B. As can be seen from the table, the coefficient of DOI is 0.003 and still significant at the 1% level, indicating that the experimental results remain robust after replacing the explanatory variables.

#### Intercept subsamples

Considering that the shock of the 2008 financial crisis on Chinese listed companies will have an impact on the financialization of firms and the interference of different sample structure and capacity on the findings, this paper uses the practice of Liu and Xie (2021) for reference, and only retains samples from years after 2012 for regression test, with other control variables remained unchanged, and the results are shown in Table 8 Panel C. The results show that the coefficient of DOI is still significantly positive at the 1% level after intercepting the subsamples for regression, indicating that the effect of D&O insurance on the promotion of corporate financialization still exists, which proves the robustness of the paper’s findings.

### Heterogeneity tests

#### Analysis from the perspective of different property rights

In China, enterprises with different property rights differ in terms of their business objectives, social functions, internal controls and the policy dividends they receive. The characteristics exhibited by corporate managers can also vary greatly. In order to explore the heterogeneous influence played by property rights in subscribing to D&O insurance to influence corporate financialization, this paper divides the enterprises into state-owned (SOEs) and non-state-owned (non-SOEs) groups to conduct regressions separately, and the results are shown in Table 9. The results show that subscription to D&O insurance still has a significant contribution to corporate financialization in the state-owned group, but not in the non-state-owned group.

**Table 9:** Sample regression results of different property rights.

	SOEs:*Fin*	non-SOE:*Fin*
*DOI*	0.007^***^	0.003
	(3.51)	(1.29)
*Cons*	0.048^***^	0.053^***^
	(6.29)	(6.92)
Control variables	control	control
Year	control	control
Industry	control	control
*N*	11,518	16,713
*R^2^*	0.1985	0.1210
*F*	69.32	54.63

*p < 0.1;

**p < 0.05;

***p < 0.01.

#### Analysis from the perspective of different life cycle stages

Corporate life cycle theory suggests that the operational, governance, and investment decisions taken by firms at different life cycle stages will exhibit different characteristics. Guan et al. (2021) found that the effect of D&O insurance on the inefficient investment of a firm will show different characteristics depending on the life cycle stages. Then, will the promotion of D&O insurance on the financialization of enterprises be different due to different life cycles stage of enterprises? This paper draws on the cash flow method used by Liu et al. (2020) to classify the enterprise life cycle, and explores whether heterogeneous characteristics may exist in the effect of subscribing to D&O insurance on corporate financialization across different life cycle stages. Listed enterprises are divided into three major stages: growth, maturity and decline, and the regression results are shown in Table 10. The table shows that the effect of subscription to D&O insurance on financialization is still significantly positive in growth and decline stage firms, but not in maturity stage firms.

**Table 10:** Sample regression results of different life cycle stages.

	Growth stage: *Fin*	Maturity stage: *Fin*	Decline stage: *Fin*
*DOI*	0.006^***^	0.002	0.010^**^
	(2.63)	(0.76)	(2.13)
*Cons*	0.043^***^	0.046^***^	0.074
	(6.32)	(5.17)	(4.78)
Control variables	control	control	control
Year	control	control	control
Industry	control	control	control
*N*	12,106	10,634	5,491
*R^2^*	0.1356	0.1443	0.1403
*F*	45.04	42.52	21.16

*p < 0.1;

**p < 0.05;

***p < 0.01.

#### Analysis from the perspective of CEO duality

The leadership structure of a company’s board of directors can have a very important impact on the internal control of the company. The CEO duality means that CEO also serves as chairman of the board of directors, which may lead to high decision efficiency because of high concentration of power and also results in moral hazard due to the weakened supervisory role of the board of directors. Therefore, this paper further analysis the results obtained above from the perspective of CEO duality, and the regression results are shown in Table 11. The regression results show that the contribution of subscribing to D&O insurance to corporate financialization is not significant in firms with CEO duality, while the results in firms with no CEO duality remain consistent with the previous paper.

**Table 11:** Sample regression results of whether the chairman and general manager are united in two positions.

	Two positions in one:*Fin*	Not two positions in one:*Fin*
*DOI*	0.004	0.006^***^
	(0.90)	(3.63)
*Cons*	0.046^***^	0.050^***^
	(3.68)	(8.44)
Control variables	control	control
Year	control	control
Industry	control	control
*N*	7,045	21,186
*R^2^*	0.1235	0.1525
*F*	23.49	90.60

* *p* < 0.1;

** *p* < 0.05;

*** *p* < 0.01.

## Discussions

This study demonstrates that there is a positive relationship between subscription to D&O insurance and corporate financialization. This implies that, as described in the previous theoretical section of this paper, subscription to D&O insurance enhances firms’ willingness to allocate financial assets. In terms of the influential mechanism, subscription to D&O insurance promotes financialization by increasing risk-taking, alleviating financing constraints, and improving audit quality. Based on the perspective of insurance contracts, this paper confirms that D&O insurance exacerbates the tendency of financialization of enterprises, supports the opportunistic hypothesis, and further provides empirical evidence and practical reference for avoiding excessive outflow of funds from the real economy to fictitious economy. The establishment of an explanatory mechanism for the relationship between D&O insurance and corporate financial asset allocation helps to gain a deeper understanding of the underlying logic of the insurance contracts exacerbating the tendency of real enterprises to shift from real to virtue, and enriches the research on the relationship between insurance governance and corporate behavior. The above findings help to understand the role of D&O insurance in corporate governance and provide important insights for regulators, listed companies and insurance companies to further improve corporate governance of the real enterprises, and optimize corporate behavior, and ultimately promote the healthy development of the real economy.

In addition, this paper also tested for heterogeneity in its effect by means of subsample regression. The results in the heterogeneity analysis suggest that the promotion of financialization by subscribing to D&O insurance is more significant in state-owned enterprises, growth and decline stage enterprises, and non-dual-employment enterprises. What follows are discussions of the emergence of such heterogeneous results in this paper.

### Heterogeneous discussion of different property rights nature

The reasons for the heterogeneity of the impact of subscribing to D&O insurance on corporate financialization among companies with different property rights nature are as follows. The first reason is that in China, state-owned enterprises usually face less financing constraints, and usually have more redundant funds or have more opportunities to obtain lower cost funds in the market, thus providing enterprises with greater capital convenience for financial asset investment. In contrast, non-state-owned enterprises usually face strong financing constraints. After obtaining scarce funds, enterprises usually use them in the most urgent or valuable projects. The second reason is that the state-owned enterprises have some problems of empty property rights, and managers have stronger control power. In order to obtain higher self-interest and strengthen their positions, senior executives of state-owned enterprises have a stronger motivation to obtain higher short-term interests for enterprises to earn high rewards for themselves. In contrast, the shareholders of non-state-owned enterprises will have stronger control over managers, and enterprises will pay more attention to the maximization of shareholder value, so as to invest in projects with long-term value. Therefore, subscription of D&O insurance has a more significant impact on enterprise financialization in state-owned enterprises.

### Heterogeneous discussion of different life cycle stages

The reason for the heterogeneous result among firms at different life cycle stages may be that when the enterprise enters the mature stage, they mature in all aspects and can achieve maximum business performance in exchange for minimum operating costs (Durana et al., 2021). Their governance mechanism tends to be improved, and the possibility of senior executives’ mistakes or misjudgments becomes smaller, so it is not necessary to rely too much on the D&O insurance to provide guarantee and incentive for the enterprising behavior of senior executives. At the same time, under the matured governance mechanism, the opportunistic behavior of senior executives will also be strictly controlled, and the opportunistic behavior of improving personal interests through financialization will also be restrained. The enterprises that subscribe for D&O insurance in the mature stage cannot significantly promote the enterprise financialization.

### Heterogeneous discussion of different CEO duality

The reason for the heterogeneous result among firms at different CEO duality may be that the CEO of an enterprise with two positions in one has stronger control over the enterprise and is more willing to engage in strategic project investment. Because from the perspective of investors’ expectations, the stronger the control of the CEO over the enterprise, the more it should do some unique projects with long-term impact for the enterprise, rather than the financial investment that is not conducive to leaving the unique mark of the CEO for the enterprise. Therefore, the purchase of D&O insurance is usually used to stimulate the CEO’s real long-term innovation and risk-taking, rather than to spur the financialization of the enterprise.

## Conclusions and limitations

### Conclusion

This paper examines the relationship between subscription to D&O insurance and corporate investment of financial assets through empirical analysis by selecting a sample of A-share listed companies in non-financial industries from 2008 to 2020, and verifies the influence mechanism of D&O insurance on corporate financialization. It is found that subscription to D&O insurance significantly increases the degree of financialization of enterprises. The empirical results also validate the partial mediating role of risk-taking, financing constraints, and audit quality. Through further analysis, i.e., group regression on the sample of different property rights, different life cycles and different states of CEO duality, it is found that the promotion effect of subscribing to D&O insurance on corporate financialization is not significant in non-state owned enterprises, mature-staged enterprises, and enterprises with CEO duality. The conclusions of this paper help enrich the study of the economic consequences of subscribing to D&O insurance for enterprises and the factors influencing the financialization of enterprises, broadening the research field of financialization with good practical significance.

### Policy suggestions

Based on the above research, this paper proposes the following recommendations: (1) The participation rate of D&O insurance in China is still low, so the government should strengthen the formulation of laws and regulations related to D&O insurance, improve financial supervision, and actively promote the participation rate of D&O insurance, so as to reduce the business risks brought to enterprises by executive decision-making errors. (2) Enterprises should actively respond to the call of the national policy and take out D&O insurance, so as to give play to the incentive and supervision effect brought by the subscription of D&O insurance, and promote the healthy development of enterprises. (3) Strengthen the internal and external supervision mechanism, supervise the decision-making behavior of senior executives from shareholders, insurance companies and the government, and prevent moral hazard and opportunistic behavior caused by D&O insurance. (4) Listed companies should improve internal corporate governance, reduce the opportunistic behavior of executives that may occur as a result of subscribing to D&O insurance, and prevent excessive financialization of enterprises. (5) Listed companies should reasonably allocate financial assets, optimize the asset structure of the enterprise, and timely adjust the ratio of financial assets, taking into account the capital status and strategic objectives of the enterprise, so as to create good opportunities for the long-term development of the enterprise.

### Limitations and future research

There are still some limitations in this paper. First of all, the study in this paper is based on a sample of Chinese firms and the conclusions drawn may not be applicable to other countries. It will be interesting to compare China with other countries for a more in-depth study if in the future we can get information about the subscription of D&O insurance and the allocation of financial assets by companies in other countries. By reviewing the relevant information, some other countries, such as the United States, South Korea, and Germany, have been deepening the financialization of enterprises, and the number of enterprises subscribing to D&O insurance in these countries has a certain scale. So, will the impact of subscription to D&O insurance on corporate financialization in these countries be the same as in China, or does it behave differently? What are the reasons for the differences? This is all worth studying in the future. Second, due to the difficulty of data acquisition, this paper only considers whether enterprises subscribe to D&O insurance without considering the subscription amount when analyzing the situation of enterprises subscribing to D&O insurance, which is not conducive to considering the real situation of enterprises subscribing to D&O insurance from a more comprehensive perspective. In the future, we will further enhance the data collection and re-test the amount of D&O insurance subscription as an explanatory variable to provide a valid addition to this study. Finally, this paper considers the impact of subscribing directors’ and officers’ liability insurance on corporate financialization, which can be enriched in the future by considering the impact of other characteristics of the enterprises on it.

## Data availability statement

Publicly available datasets were analyzed in this study. This data can be found at: the Chinese CSMAR database (http://cn.gtadata.com), the WIND database (www.wind.com.cn) and the CNRDS database (www.cnrds.com).

## Author contributions

All authors listed have made a substantial, direct, and intellectual contribution to the work and approved it for publication.

## Funding

This study was funded by Chongqing Federation of Social Sciences, “The research on the impact of accounting standards difference on the two-way interactive investment between Chongqing and the ‘the Belt and the Road’” (Grant no. 2018YBJJ032). Sichuan International Studies University, “The theoretical and empirical research on the influence of environmental information disclosure on firms’ green preferences for foreign investment” (Grant no. SISU2022YZ009). Chongqing Education Commission, “The research on the impact of digital transformation on cross-border M&A: From the perspective of M&A motivation” (Grant no. CYS22519).

## Conflict of interest

The authors declare that the research was conducted in the absence of any commercial or financial relationships that could be construed as a potential conflict of interest.

## Publisher’s note

All claims expressed in this article are solely those of the authors and do not necessarily represent those of their affiliated organizations, or those of the publisher, the editors and the reviewers. Any product that may be evaluated in this article, or claim that may be made by its manufacturer, is not guaranteed or endorsed by the publisher.

## References

[ref1] Bulathsinhalage S. Pathirawasam C. (2017). The effect of corporate governance on firms’ capital structure of listed companies in Sri Lanka. J. Compet. 9, 19–33. doi: 10.7441/joc.2017.02.02

[ref2] Chen Z. H. Li Z. O. Zou H. (2016). Directors’ and officers’ liability insurance and the cost of equity. J. Account. Econ. 61, 100–120. doi: 10.1016/j.jacceco.2015.04.001

[ref3] Chen H. Wang S. H. Liu L. F. Hu Y. D. (2021). Does directors' executive liability insurance promote corporate outbound direct investment. Statist. Informat. Forum 36, 107–117.

[ref4] Dai B. Wang J. Y. (2021). Internationalization of the board of directors and financialization of the firm: “rigid” or "flexible". Res. Financ. Econ. 36, 124–141.

[ref5] Du Y. Zhang H. Chen J. Y. (2017). The impact of financialization on the future development of real enterprises’ main business: facilitating or inhibiting. Chin. Indust. Econ. 113–131. doi: 10.19581/j.cnki.ciejournal.20171214.007

[ref6] Duong C. D. Le T. L. Ha N. T. (2021). The role of trait competitiveness and entrepreneurial alertness in the cognitive process of entrepreneurship among students: a cross-cultural comparative study between Vietnam and Poland. J. Competit. 13, 25–42. doi: 10.7441/joc.2021.04.02

[ref7] Durana P. Michalkova L. Privara A. Marousek J. Tumpach M. (2021). Does the life cycle affect earnings management and bankruptcy? Oeconomia Copernicana 12, 425–461. doi: 10.24136/oc.2021.015

[ref8] Durana P. Valaskova K. Vagner L. Zadnanova S. Podhorska I. Siekelova A. (2020). Disclosure of strategic managers’ factotum: behavioral incentives of innovative business. Int. J. Financ. Stud. 8:17. doi: 10.3390/ijfs8010017

[ref9] Feng L. Q. Kong X. T. Cao H. J. (2017). Directors’ executive liability insurance and the cost of equity capital - evidence from an empirical study of the information quality channel. Account. Res. 65–71+97.27753509

[ref10] Gao T. Zhang Y. X. Xu H. P. (2021). Directors’ executive liability insurance and corporate internal control quality: empirical evidence based on A-share listed companies. Financ. Regul. Res. 33–48. doi: 10.13490/j.cnki.frr.2021.05.003

[ref11] Gu L. L. Guo J. L. Wang H. Y. (2020). Corporate social responsibility, financing constraints and corporate financialization. Financ. Stud. 109–127.

[ref12] Gu H. F. Zhang H. H. (2022). How does corporate financialization affect the efficiency of real investment? Based on evidence from Chinese A-share listed companies. J. Manag. 35, 86–101. doi: 10.19808/j.cnki.41-1408/f.2022.0007

[ref13] Guan X. Chai C. J. Gao B. G. (2021). The inhibitory mechanism of directors' liability insurance on firms' inefficient investment – based on the co-mediation of monitoring and signaling effects. Econ. Manag. Stud. 42, 93–112. doi: 10.13502/j.cnki.issn1000-7636.2021.12.008

[ref14] Guo F. Ma R. Xie X. B. (2022). Industrial policy, business environment and enterprises from virtual to real: empirical evidence based on the national five-year plan. J. Financ. Econ. 48, 33–46. doi: 10.16538/j.cnki.jfe.20211114.301

[ref15] Hu J. Dai M. Hu Q. Q. (2019). Directors' and Officials' liability insurance and corporate business credit. Finan. Theory Pract. 40, 62–68. doi: 10.16339/j.cnki.hdxbcjb.2019.05.030

[ref16] Hu G. L. Hu J. (2017). D&O liability insurance and corporate risk-taking: theoretical path and empirical evidence. Account. Res. 40-46+96.

[ref17] Hu G. L. Li Y. Zhao Y. (2020). Directors' executive liability insurance and the demand for high quality audit services for companies. Audit Res. 97–105.

[ref18] Hu G. L. Tan L. (2018). Directors' and Officials' liability insurance and credit rating: an empirical analysis based on China's A-share listed companies. Insur. Res. 81–92. doi: 10.13497/j.cnki.is.2018.09.008

[ref19] Jensen M. C. Meckling W. H. (1976). Theory of the firm: managerial behavior, agency costs and ownership structure. J. Financ. Econ. 3, 305–360. doi: 10.1016/0304-405X(76)90026-X

[ref20] Krulicky T. Horak J. (2021). Business performance and financial health assessment through artificial intelligence. Ekonomicko-manazerske Spektrum 15, 38–51. doi: 10.26552/ems.2021.2.38-51

[ref21] Lei X. Tang X. S. Jiang X. Y. (2020). Can directors' executive liability insurance curb corporate irregularities? Econ. Manag. Stud. *02*, 127–144. doi: 10.13502/j.cnki.issn1000-7636.2020.02.009

[ref22] Li X. Tong Y. Zong K. (2021). Management equity incentives and Financialization of entity enterprises. J. Beijing Technol. Bus. Univ. 36, 54–66.

[ref23] Liu S.Y. Lin C.F. Leng C.P. (2020). Do tax incentives improve firm innovation?--A test based on firm life cycle theory. Econ. Res. 55, 105–121

[ref24] Liu Z. W. Liu J. Q. Yang Y. Yang S. G. (2019). Corporate social responsibility and corporate Financialization: Financial instruments or management tools? Account. Res. 57–64.

[ref25] Liu M. K. Xie X. B. (2021). Corporate financialization, financing constraints and sustainable growth. Southern Finan. 38–50.

[ref26] Liu J. J. Zhang D. N. Li H. Z. (2022). A study of financialization and industrial investment of Chinese listed companies – a reexamination of the motives and regulatory effects of financialization. Manag. Rev. 34, 37–50. doi: 10.14120/j.cnki.cn11-5057/f.2022.01.005

[ref27] Ma Y. M. Yang L. (2022). Strict financial supervision, corporate financialization and capital allocation efficiency of real economy. Finan. Trade Res. 33, 40–52. doi: 10.19337/j.cnki.34-1093/f.2022.01.004

[ref28] Mitan A. Siekelova A. Rusu M. Rovnak M. (2021). Value-based management: a case study of Visegrad four countries. Ekonomicko-manazerske spektrum 15, 87–98. doi: 10.26552/ems.2021.2.87-98

[ref29] Narjess B. Georges D. Thouraya T. (2008). Consolidation and value creation in the insurance industry: the role of governance. J. Bank. Finan. 32, 56–68. doi: 10.1016/j.jbankfin.2007.09.004

[ref30] Peng Y. C. Han X. Li J. J. (2018). Economic policy uncertainty and corporate financialization. China's Industr. Econ. 137–155. doi: 10.19581/j.cnki.ciejournal.20180115.010

[ref31] Tang X. S. Liao W. Liao Q. (2021). Does the design of insurance system affect the internationalization strategy of companies: Evidence from D&O liability insurance. Contemp. Finance Econ. 77–89. doi: 10.13676/j.cnki.cn36-1030/f.2021.12.008

[ref32] Tijani A. A. Osagie R. O. Afolabi B. K. (2021). Effect of strategic alliance and partnership on the survival of MSMEs post COVID-19 pandemic. Ekon. Manazerske Spektrum 15, 126–137. doi: 10.26552/ems.2021.2.126-137

[ref33] Valaskova K. Adamko P. Frajtova Michalikova K. Macek J. (2021b). Quo Vadis, earnings management? Analysis of manipulation determinants in central European environment. Oeconomia Copernicana 12, 631–669. doi: 10.24136/oc.2021.021

[ref34] Valaskova K. Androniceanu A.-M. Zvarikova K. Olah J. (2021a). Bonds between earnings management and corporate financial stability in the context of the competitive ability of enterprises. J Competitiveness 13, 167–184. doi: 10.7441/joc.2021.04.10

[ref35] Valaskova K. Kliestik T. Gajdosikova D. (2021c). Distinctive determinants of financial indebtedness: evidence from Slovak and Czech enterprises. Equilibrium 16, 639–659. doi: 10.24136/eq.2021.023

[ref300] Wan L. Y. Zha Y. Y. Rao J. (2011). A new genus of the genus A (Coleoptera, Staphylinidae, Staphylininae) from China (2020): financialization of real firms and firms’ innovation output-with moderating mediating effects. Account. Res. 98–111.

[ref36] Wang W. Hu Z. (2022). The impact of technical background CEOs on corporate financialization: a regulated mediation model. Sci. Technol. Progr. Countermeasures 39, 142–152.

[ref37] Wang Y. H. Zhang T. (2014). Financial innovation, audit quality and bank risk tolerance – empirical evidence from commercial banks in China. Account. Res. 81–87+96.

[ref38] Wen W. (2017). Directors' and officials' liability insurance for and corporate risk assumption. J. Shanxi Univ. Finan. Econ. 39, 101–112. doi: 10.13781/j.cnki.1007-9556.2017.08.008

[ref39] Xing F. Zhou T. Y. (2020). Directors' executive liability insurance and corporate strategy. Insur. Res., 32–46. doi: 10.13497/j.cnki.is.2020.11.003

[ref40] Xu J. Liu F. (2020). The impact of intellectual capital on firm performance: a modified and extended VAIC model. J. Competitiveness 12, 161–176. doi: 10.7441/joc.2010.01.10

[ref41] Xu J. C. Zeng X. Y. (2011). A new species of the genus Phyllostachys (Hymenoptera, Braconidae) from China. (2010). Fair value measurement and management compensation covenants. Account. Res.:12–19+96.

[ref42] Yang Z. Wang H. J. Dai J. Xu C. H. (2019). Deregulation of interest rates, equality of rate of profit and the “derealization of real to virtual” of entity enterprises. Financial Res. 20–38.

[ref43] Ye C. G. Chu C. H. Duan C. M. (2020). Media attention, auditor change and audit quality. J. Xi'an Univ. Finance Econ. 33, 39–48. doi: 10.19331/j.cnki.jxufe.2020.06.005

[ref44] Yu M. G. Li W. G. Pan H. B. (2013). Privatization, property rights protection and corporate risk-taking. Econ. Res. 48, 112–124.

[ref45] Yuan R. L. Li R. J. Li B. X. (2018). Directors' executive liability insurance and audit fees. Audit Res. 55–63.

[ref46] Zeng C. H. Li Y. (2018). Does director executive liability insurance increase a company's audit fees? Audit Econ. Res. 33, 46–54.

[ref47] Zhang J. H. Chang Q. G. Yin Y. F. (2021). Whether tax incentives can inhibit corporate financialization: a quasi-natural experiment based on accelerated depreciation of fixed assets. Financial Regulat. Res. 39–55. doi: 10.13490/j.cnki.frr.2021.12.004

[ref48] Zhang Z. L. Xu F. Y. (2021). Directors’ and officers' liability insurance and high-quality development of enterprises: based on agency costs and innovation incentive perspectives. East China Econ. Manag. 78–86. doi: 10.19629/j.cnki.34-1014/f.200911006

[ref49] Zhang D. R. Zhao S. Z. (2022). Managerial behavior, internal and external monitoring, and corporate financialization. Stud. Finance Econ. 121–128. doi: 10.19654/j.cnki.cjwtyj.2022.04.013

[ref50] Zheng D. J. Yan T. Y. (2016). Accounting robustness, audit quality and cost of debt. Audit. Res. 74–81.

